# The relationships between thyroid-stimulating hormone level and insulin resistance, glucose effectiveness, first- and second-phase insulin secretion in Chinese populations

**DOI:** 10.1097/MD.0000000000025707

**Published:** 2021-05-14

**Authors:** Tsung-Ju Chuang, Jiunn-Diann Lin, Chung-Ze Wu, Hui-Chun Ku, Chun-Cheng Liao, Chih-Jung Yeh, Dee Pei, Yen-Lin Chen

**Affiliations:** a Division of Endocrinology and Metabolism, Department of Internal Medicine, Taichung Armed Forces General Hospital, Taichung, National Defense Medical Center, Taipei, Taiwan; b Division of Endocrinology and Metabolism, Department of Internal Medicine, Shuang Ho Hospital; c Division of Endocrinology, Department of Internal Medicine, Shuang Ho Hospital; d College of Medicine, Taipei Medical University, Taipei, Taiwan, ROC; e Department and Institute of Life Science, Fu-Jen Catholic University, New Taipei City; f Department of Family Medicine, Taichung Armed Forces General Hospital, Taichung, National Defense Medical Center, Taipei; g School of Public Health, Chung Shan Medical University, Taichung; h Division of Endocrinology and Metabolism, Department of Internal Medicine, Fu-Jen Catholic Hospital, Fu Jen Catholic University, School of Medicine, New Taipei City; i Department of Pathology, Cardinal Tien Hospital, Fu Jen Catholic University, School of Medicine, New Taipei City, Taiwan, ROC.

**Keywords:** diabetes mellitus, first phase insulin secretion, glucose effectiveness, insulin resistance, second phase insulin secretion, thyroid-stimulating hormone

## Abstract

Increased insulin resistance (IR); decreased glucose effectiveness (GE); and both first-and second phase of insulin secretion (FPIS, SPIS) have always been important factors for the development of type 2 diabetes. Therefore, in this study, we evaluated the relationships between thyroid-stimulating hormone (TSH) and these 4 factors in adult Chinese.

We randomly enrolled 24,407 men and 24,889 women between 30 and 59 years old. IR, FPIS, SPIS and GE were measured with the equations built by our group.

IR = log (1.439 + 0.018 × sex - 0.003 × age + 0.029 × BMI - 0.001 × SBP + 0.006 × DBP + 0.049 × TG - 0.046 × HDLC - 0.0116 × FPG) × 10 ^3.333^
FPIS = 10 [1.477 - 0.119 × FPG + 0.079 × BMI - 0.523 × HDLC]
SPIS = 10 [-2.4 - 0.088 × FPG + 0.072 × BMI]
GE = (29.196 - 0.103 × age - 2.722 × TG - 0.592 × FPG) ×10 ^−3^

The *t* test was performed to evaluate the differences between normal and diabetic groups. To evaluate the differences of the mean values of the 4 groups, from the highest to the lowest levels of TSH, we used a one-way analysis of variance.

Age, high density lipoprotein-cholesterol and GE were higher in women. On the other hand, body mass index, blood pressure, low density lipoprotein-cholesterol, triglyceride, FPIS, SPIS and IR were higher in men. TSH was positively related to IR, FPIS, and SPIS and negatively related to GE. According to the *r* values, the tightest relationship was between TSH and IR, followed by GE, FPIS and SPIS.

In conclusion, our data showed that IR, FPIS, and SPIS were positively related to the TSH level in middle-aged Chinese, whereas GE was negatively related. In both genders, IR had the tightest association followed by GE, FPIS, and SPIS.

## 1 Introduction

In the past 2 decades, the prevalence of diabetes increased drastically in Taiwan as well as many other countries. As shown in a recent study, among those aged between 20 to 79 years, its prevalence has reached 6.38%.^[1]^

One of the many possible causes that explain this phenomenon is the westernization of lifestyle which has led to obesity among the general population.^[2]^ This trend has already brought a heavy burden not only to the individual but also to the healthcare providers and the government. Unfortunately, the underlying pathophysiology of type 2 diabetes (T2D) is still not completely clear. There are 4 main factors which are known to contribute glucose tolerance. When these factors are deteriorating, diabetes will ensue. The first one is insulin resistance (IR) which refers to the blunting of insulin action in muscle, liver and fat tissue.^[3]^ The second one is insulin secretion. However, it should be noted that there are 2 phases. The first phase (FPIS: first phase insulin secretion) lasts for 10 minutes immediately after the challenge of glucose in any form. It is the release of the insulin stored in the vesicles of the pancreas. In the same time, the newly produced insulin after the first 10 minutes is considered to be the second phase (SPIS).^[4]^ Finally, glucose has the ability to eliminate itself independent of the insulin action which is termed as glucose effectiveness (GE).^[5]^ It is interesting that some researchers think that it is even more important than the aforementioned 3 factors, in the present study, they were denoted as diabetes factor (DF).

It is interesting to note that thyroid disease is closely related to diabetes.^[6]^ Both hyper- and hypothyroidism were reported to be related to the risk factors for glucose intolerance. The underlying pathophysiology is complicated and remains unknown. For instance, triiodothyronine has been found to be related to the preservation of beta-cell viability and its proliferation thus reducing the chance to have diabetes^[7,8]^ On the contrary, evidence has also shown that thyrotoxicosis (high thyroxine, triiodothyronine and low thyroid stimulating hormone, TSH) has a deleterious effect on glucose metabolism.^[9]^ Thus, the exact roles of thyroid hormone on glucose metabolism are of interest and need further investigation. In the present study, we enrolled 49,296 non-diabetic, non-obese and middle age healthy adults and measured their DFs by using the equations developed by our group. Our goal was to explore the exact effects of thyroid stimulating hormone on DFs.

## 2 Methods

### 2.1 Study subjects

This study was approved by institutional review board of Tri-Service General Hospital (TSGH), Cardinal Tien Hospital (CTH) and the MJ Health Management Institution. TSGH is a medical center in northern part of Taiwan. At the same time, CTH is a district hospital. Finally, MJ Health Management Institution is a large health examine center and has provided health screening services for over 1 million persons. Written informed consents were obtained from all participants, and the study protocol was approved by the institutional review board of the institutions. Data were recorded in anonymous form and information related to the identification of individuals was removed.

We randomly enrolled 24,407 men and 24,889 women between 30 to 59 years of age and who underwent routine health exams at MJ Health Screening Center in Taiwan between 2011 and 2012. MJ Health Screening Centers are private chain-clinics that provide regular health examinations for their members.

All study participants were anonymous and gave informed consent and the data were provided for research purposes only. Participants who take drugs for hypertension, hyperlipidemia, and hyperglycemia were excluded.

We further divided participants into 4 groups according to the quartiles of TSH level in order to observe the effect of TSH on DFs.

On the day of the study, senior nursing staff obtained subjects’ medical history including current medications via questionnaire and then implemented a complete physical examination. Body mass index (BMI) was calculated by dividing the subject's body weight (kg) with the square of the subject's height (m) whereas Waist Circumference (WC) was measured horizontally at the level of the natural waist. Systolic blood pressure (SBP) and diastolic blood pressure (DBP) were measured by nursing staff using standard mercury sphygmomanometers on the right arm of each subject when seated.

After the subject had fasted for 10 hours, blood samples were drawn from the antecubital vein for biochemical analysis. The plasma used for analyzing fasting plasma glucose (FPG) and lipid profiles was separated from the blood within 1 hour before being measured. FPG was measured using a glucose oxidase method (YSI 203 glucose analyzer, Yellow Springs Instruments, Yellow Springs, USA). Total cholesterol and triglycerides (TG) were measured using a dry, multilayer analytical slide method with the Fuji Dri-Chem 3000 analyzer (Fuji Photo Film, Tokyo, Japan). Serum high-density lipoprotein cholesterol (HDL-C) and low-density lipoprotein cholesterol (LDL-C) concentration were analyzed using an enzymatic cholesterol assay following dextran sulfate precipitation. The level of TSH was quantified by chemiluminescence with Simens Advia CentrauXP (Irland).

We used the equations taken from our study groups listed below (in international units) to quantify the DFs. To demonstrate the reliability of our equations, a short statement is given here. Approximately, we used 70% of the data from the participants to build the equation while the remaining 30% were used for external validation. Thus, the accuracy of the equations could be tested.

1. IR: We enrolled 327 subjects to measure the IR by using insulin suppression test. The *r* value between the measured and calculated GE was 0.581 (*P* < .001). It was published in “Journal of Diabetes Investigation” in 2013.
  IR = log (1.439 + 0.018 × sex - 0.003 × age + 0.029 × BMI - 0.001 × SBP + 0.006 × DBP + 0.049 × TG - 0.046 × HDLC - 0.0116 × FPG) × 10 ^3.333^^[10]^
2. FPIS: We enrolled 186 subjects and measured the FPIS by using frequent sampled intravenous glucose tolerance tests. The r value between the measured and calculated GE was 0.671 (*P* < .000).
  FPIS = 10 [1.477 - 0.119 × FPG + 0.079 × BMI - 0.523 × HDLC] ^[11]^
3. SPIS: We enrolled 82 participants to measure the SPIS by using a modified low dose glucose infusion test. The *r* value between the measured and calculated GE was 0.65 (*P* = .002).
  SPIS = 10 [-2.4 - 0.088 × FPG + 0.072 × BMI] ^[12]^
4. GE: We enrolled 227 participants to measure the GE by usng frequent sampled intravenous glucose tolerance tests. The r value between the measured and calculated GE was 0.43 (*P* = .001).
  GE = (29.196 - 0.103 × age - 2.722 × TG - 0.592 × FPG) ×10 ^−3^^[13]^

### 2.2 Statistical analysis

We performed all statistical analyses using SPSS 19.0 (IBM Inc., Armonk, New York). Data were presented as mean ± standard deviation. All data were tested for normal distribution with the Kolmogorov–Smirnov test and for homogeneity of variances with Levene test. The data of FPIS, SPIS, and TG were log transformed before analysis. The *t* test was performed to evaluate the differences between normal and diabetic groups. To evaluate the differences of the mean values of the 4 groups, from the highest to the lowest levels of TSH, we used a one-way analysis of variance. Bonferroni was chosen as the postHoc method for comparison between groups.

We applied simple correlation to evaluate the relationships of 2 independent variables such as TSH and IR and the slopes of these relationships could also be obtained. The *r* values of these relationships could be regarded as a correlation coefficient. Among these 4 factors, only the GE had a negative correlation while TSH had a positive one with a higher value. In order to compare the slope of GE with the other 3 factors, we plotted a mirror-line (or reciprocal) from the 4th quadrant to the 1st quadrant but with the same slope.

## 3 Results

The demographic data and parameters of men and women were shown in Table 1. Age, HDL-cholesterol and GE were higher in women while BMI, blood pressure, LDL-cholesterol, TG, FPIS, SPIS, and IR were higher in men. Table 2 depicts the demographical, clinical and biochemical data in TSH quartiles. The main purpose was to evaluate the change of trend in these data. Not surprisingly, similar to table 1, a similar trend could be noted for the BMI, blood pressure, LDL-C, TG and DF which showed a significant increase. On the other hand, HDL-C had the opposite increasing trend. In Table 3, the results of simple correlation between TSH and the 4 DFs are depicted. The higher the *r* value, which is also the correlation coefficient, the tighter the correlation between the 2 parameters. It can be noted that IR, FPIS, and SPIS were all positively correlated with TSH in both genders. However, GE was the only factor negatively correlated with TSH.

**Table 1:** The demographic data, clinical and biochemical parameters of subjects.

	Male	Female	*P*
n	24407	24889	
Age (year)	41.5 ± 8.3	42.8 ± 8.7	<.001
Body mass index (kg/m^2^)	24.1 ± 2.9	23.1 ± 3.2	<.001
Systolic blood pressure (mm Hg)	118.7 ± 13.8	114.8 ± 15.3	<.001
Diastolic blood pressure (mm Hg)	75.6 ± 10.2	72.3 ± 12.1	<.001
HDL-C (mg/dL)	1.1 ± 0.3	1.3 ± 0.3	<.001
LDL-C (mg/dL)	3.4 ± 0.9	3.3 ± 0.8	<.001
TG (mg/dl)	1.6 ± 0.9	1.2 ± 0.7	<.001
TSH (U_/mL)	1.4 ± 0.8	1.6 ± 0.9	<.001
FPIS (μU/min)	197.4 ± 204.6	136.9 ± 171.9	<.001
SPIS (pmol/mmol)	0.082 ± 0.065	0.073 ± 0.069	<.001
IR (10^−4^ · min^−1^ · pmol^−1^ · L^−1^)	3.700 ± 0.025	3.688 ± 0.024	<.001
GE (10^−2^ · dL · min^−1^ · kg^−1^)	0.017 ± 0.003	0.018 ± 0.002	<.001

FPIS = first phase insulin secretion, GE = glucose effectiveness, HDL-C = high-density lipoprotein cholesterol, IR = insulin resistance, LDL-C = low-density lipoprotein cholesterol, SPIS = second phase insulin secretion, TG = triglyceride, TSH = thyroid stimulating hormone. Data are shown mean ± SD.

**Table 2:** The demographic data, clinical and biochemical parameters in quartiles of thyroid stimulation hormone.

	TSH tertile 1	TSH tertile 2	TSH tertile 3	TSH tertile 4	*P*
Male					
n	6169	5820	6073	6345	
Age (yr)	42.3 ± 8.6^234^	41.3 ± 8.3^1^	41.2 ± 8.2^1^	41.4 ± 8.2^1^	<.001
Body mass index (kg/m^2^)	24.0 ± 2.8^4^	24.0 ± 2.9	24.2 ± 3.0	24.2 ± 3.0^1^	<.001
Systolic blood pressure (mm Hg)	118.1 ± 14.0^4^	118.3 ± 13.7^4^	118.7 ± 13.5	119.6 ± 13.7^12^	<.001
Diastolic blood pressure (mm Hg)	74.8 ± 10.2^34^	75.3 ± 10.1^4^	75.7 ± 10.1^14^	76.6 ± 10.2^123^	<.001
HDL-C (mg/dl)	1.088 ± 0.323	1.093 ± 0.319	1.079 ± 0.323	1.081 ± 0.320	.055
LDL-C (mg/dl)	3.387 ± 0.859	3.420 ± 0.853	3.421 ± 0.856	3.431 ± 0.868	.025
TG (mg/dL)	1.520 ± 0.854^34^	1.549 ± 0.857^4^	1.595 ± 0.900^14^	1.677 ± 0.948^123^	<.001
TSH (U/_mL)	0.682 ± 0.190^234^	1.105 ± 0.100^134^	1.503 ± 0.142^124^	2.448 ± 0.665^123^	<.001
FPIS (μU/min)	188.6 ± 170.1^34^	188.7 ± 174.2^34^	205.4 ± 239.6^12^	206.1 ± 223.3^12^	<.001
SPIS (pmol/mmol)	0.079 ± 0.049^4^	0.081 ± 0.057	0.084 ± 0.068	0.084 ± 0.082^1^	<.001
IR (10^−4^ · min^−1^ · pmol^−1^ · L^−1^)	3.697 ± 0.024^34^	3.699 ± 0.024^4^	3.700 ± 0.026^14^	3.702 ± 0.026^123^	<.001
GE (10^−2^ · dL · min^−1^ · kg^−1^)	0.017 ± 0.003^4^	0.017 ± 0.003^4^	0.017 ± 0.003^4^	0.017 ± 0.003^123^	<.001
Female					
n	6274	6233	6262	6120	
Age (yr)	43.5 ± 9.0^23^	42.5 ± 8.5^1^	42.4 ± 8.5^1^	42.9 ± 8.5	<.001
Body mass index (kg/m^2^)	23.1 ± 3.1	23.0 ± 3.1^4^	23.0 ± 3.2^4^	23.3 ± 3.3^23^	<.001
Systolic blood pressure (mm Hg)	114.9 ± 15.4	114.2 ± 15.1^4^	114.7 ± 15.3	115.7 ± 15.4^2^	<.001
Diastolic blood pressure (mmHg)	71.9 ± 10.2^4^	72.0 ± 14.1^4^	72.3 ± 13.1	73.1 ± 10.4^12^	<.001
HDL-C (mg/dl)	1.306 ± 0.350^4^	1.297 ± 0.342	1.306 ± 0.347^4^	1.281 ± 0.343^13^	<.001
LDL-C (mg/dl)	3.251 ± 0.827	3.258 ± 0.814	3.284 ± 0.831	3.291 ± 0.848	.018
TG (mg/dL)	1.128 ± 0.638^4^	1.125 ± 0.629^4^	1.162 ± 0.662^4^	1.230 ± 0.711^123^	<.001
TSH (U/_mL)	0.656 ± 0.226^234^	1.163 ± 0.126^134^	1.651 ± 0.172^124^	2.809 ± 0.763^123^	<.001
FPIS (μU/min)	131.1 ± 150.6^4^	133.4 ± 163.6^4^	136.3 ± 179.6	146.9 ± 190.9^12^	<.001
SPIS (pmol/mmol)	0.072 ± 0.073	0.072 ± 0.073	0.073 ± 0.062	0.076 ± 0.070	.002
IR (10^−4^ · min^−1^ · pmol^−1^ · L^−1^)	3.687 ± 0.023^4^	3.687 ± 0.024^4^	3.688 ± 0.025^4^	3.691 ± 0.025^123^	<.001
GE (10^−2^ · dL · min^−1^ · kg^−1^)	0.018 ± 0.003^4^	0.019 ± 0.002^4^	0.019 ± 0.002^4^	0.018 ± 0.003^123^	<.001

FPIS = first phase insulin secretion, GE = glucose effectiveness, HDL-C = high-density lipoprotein cholesterol, IR = insulin resistance, LDL-C = low-density lipoprotein cholesterol, SPIS = second phase insulin secretion, TG = triglyceride, TSH = thyroid stimulating hormone. Data are shown mean ± SD, the upper case of the numbers on the left side of the values indicate there are significant difference against that particular group

**Table 3:** Results of simple correlation between thyroid stimulating hormones and the diabetes factors.

	*r*	*P*
Male		
First Phase Insulin Secretion	0.037	<.001
Second Phase Insulin Secretion	0.030	<.001
Insulin resistance	0.076	<.001
Glucose effectiveness	−0.059	<.001
Female		
First Phase Insulin Secretion	0.033	<.001
Second Phase Insulin Secretion	0.022	<.001
Insulin resistance	0.058	<.001
Glucose effectiveness	−0.049	<.001

The *r* value is the correlation coefficient derived from the simple correlation. *P* value indicates the significance is <.05.

**Figure 1:**
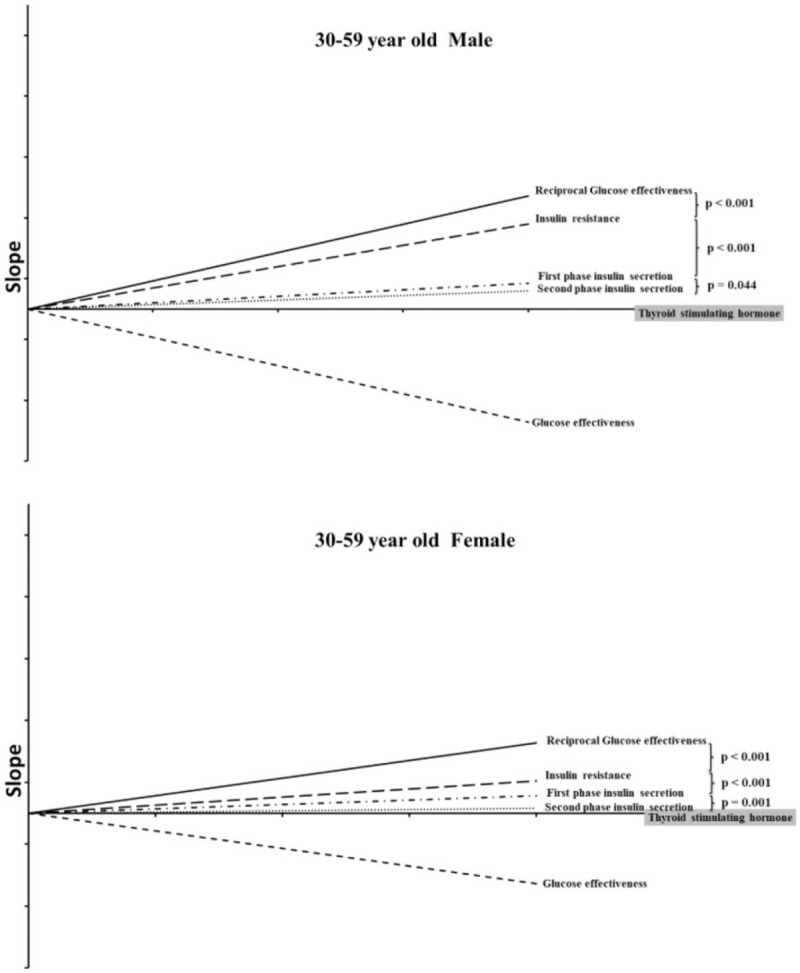
The simple correlation slope between TSH and the 4 diabetic factors.

Figure 1 is the most important finding in the present study, and it demonstrates the slopes of the 4 factors. The graphic demonstrates the relative tightness of their relationships. It was necessary to plot GE reciprocally because of its negative correlation to the level of TSH. It appears in the figure that they were all significantly related to TSH. However, the closeness of their relationships with TSH for both genders could be ranked in order of value from the highest to the lowest as follows: IR, GE, FPIS, and SPIS. It should be noted that the higher the *r* value (correlation coefficient), the tighter the relationship.

## 4 Discussion

In the present study, TSH is regarded as the indicator of thyroid function. Our data suggested in both genders IR, FPIS, and SPIS had all a positive correlation to TSH whereas GE had a negative one. Besides, the order of the tightness, from the highest to the lowest, was IR, GE, FPIS, and SPIS in non-obese and non-diabetic Chinese. This is the first study to evaluate the relations between TSH and all the 4 DFs simultaneously in the same individuals who are non-obese, non-diabetic, and middle aged healthy Chinese. This study could help us to understand the roles of TSH in glucose metabolism.

The question of whether thyroid dysfunction is related to diabetes is of interest among many researchers devoted to this area. However, until now, there is no unanimous consensus. Several cross-sectional studies show that hyperthyroidism is positively related to diabetes.^[14,15]^ On the other hand, some researchers found that T2D is associated with hypothyroidism.^[16]^ More interestingly, Fleiner et al reported a non-significant correlation between thyroid function and diabetes. Due to the fact that these are all cross-sectional studies which are less persuasive, Chaker et al performed a longitudinal study in 8452 subjects and demonstrated that hypothyroidism is a risk factor for diabetes.^[17]^ Since this study had a large number of participants and had a longitudinal design, their results should be reliable. Most importantly, their outcomes are in line with our present findings.

The relationship between TSH and IR has been studied extensively in the past. For example, Lambadiari et al reported a positive relationship between TSH and IR in T2D.^[18]^ By using the simplest method of homeostasis assessment, they found a similar result in women with polycystic ovary syndrome.^[19]^ Recently, Zue et al confirmed this relationship in non-diabetic subjects^[20]^ and our finding is also in line with the above outcomes. The possible underlying mechanisms between TSH and IR might come from the roles of adipocytes. TSH receptors are found to exist on the adipocytes.^[21]^ After binding with TSH receptors, TSH could stimulate the release of interleukin-6 from adipocytes which could further mediates its own proliferation.^[22]^ This eventually leads to the further aggravation of IR.^[23]^

Lenzen at al had showed that, in rat pancreas, lower TSH (experimental hyperthyroidism) could decrease insulin secretion. This is one of the earliest studies regarding the relationship between TSH and insulin secretion.^[24]^ After this pioneer study, very few studies were done in this area. However, some researchers suggested in their report that lower TSH is related to higher insulin secretion which is contrary to the finding of the present study.^[25]^ To explain this discrepancy, we hypothesize that the increased insulin secretion, both FPIS and SPIS, are the results of beta-cell compensation to the increased IR. In other words, the fundamental glucose dysregulation that is caused by TSH is impaired IR. The concomitant increases of FPIS and SPIS are only the beta-cell response to IR. Besides, since the age of the present study cohort was between 30 to 59 years old, it is possible that that their reserved beta-cell function was deteriorating. This could also explain that the r values between TSH and insulin secretion are relatively lower than those of the IR.

Finally, to our knowledge, there has been no study investigating the relationship between TSH and GE. The present study is the first to find a significant negative relationship between these 2 parameters. Till now, the detailed physiological and pathological roles of GE remain obscure. However, this negative relationship might be explained by inflammation. As a matter of fact, from the limited information derived from the results of Jerguson et al, it could be noted that GE decreased after the challenge of endotoxin which is an oxidative stress.^[26]^ At the same time, it is well known that hyperthyroidism is also related to oxidative stress via increased reactive oxygen species which could be regarded as inflammation markers.^[27]^ In other words, subjects with lower TSH would be potentially more prone to inflammatory processes and, thus, would have lower GE. However, this hypothesis still needs further studies in animal or cellular levels.

It should be noted that all the results were derived from relatively healthy, middle-aged subjects who had no history of significant diseases including hyperthyroidism at the time of the study. So, cautions must be taken when extrapolating our findings into hypo- or hyperthyroidism.

Our study has limitations. First, one might argue about the accuracy of the equations we used to calculate DFs. However, as we have mentioned in the methods, these equations were obtained from published well-designed studies with enough n numbers. Moreover, the large n number in the present study might justify this deficit. Secondly, this is a cross-sectional study. Compared to a longitudinal one, it is less persuasive. A longitudinal follow-up study might be valuable to further explore their relationships.

In conclusion, our data showed that IR, FPIS and SPIS were positively, and GE negatively, related to the TSH level in middle-aged Chinese. In both genders, IR had the tightest association followed by GE, FPIS, and SPIS.

## Acknowledgments

All the authors wish to thank the study assistants for their help in data preparation and collection.

## Author contributions

**Conceptualization:** Chih-Jung Yeh.

**Data curation:** Chung-Ze Wu, Chun-Cheng Liao, Yen-Lin Chen.

**Formal analysis:** Chun-Cheng Liao, Yen-Lin Chen.

**Funding acquisition:** Tsung-Ju Chuang, Yen-Lin Chen.

**Investigation:** Chih-Jung Yeh.

**Methodology:** Chih-Jung Yeh.

**Resources:** Hui-Chun Ku.

**Software:** Chung-Ze Wu, Hui-Chun Ku.

**Validation:** Dee Pei.

**Visualization:** Dee Pei.

**Writing – original draft:** Tsung-Ju Chuang, Jiunn-Diann Lin.

**Writing – review & editing:** Tsung-Ju Chuang, Dee Pei, Yen-Lin Chen.
